# Effective quantitative real-time polymerase chain reaction analysis of the parkin gene (*PARK2*) exon 1–12 dosage

**DOI:** 10.1186/1471-2350-8-6

**Published:** 2007-02-26

**Authors:** Maria I Shadrina, Elena V Semenova, Petr A Slominsky, Gulbahar H Bagyeva, Sergei N Illarioshkin, Irina I Ivanova-Smolenskaia, Svetlana A Limborska

**Affiliations:** 1 Institute of Molecular Genetics, Russian Academy of Sciences, 2 Kurchatov sq., 123182, Moscow, Russia; 2 Department of Neurogenetics, Institute of Neurology, 80 Volokolamskoye Highway, 123367, Moscow, Russia

## Abstract

**Background:**

One of the causes of Parkinson's disease is mutations in the *PARK2 *gene. Deletions and duplications of single exons or exon groups account for a large proportion of the gene mutations. Direct detection of these mutations can be used for the diagnosis of Parkinson's disease.

**Methods:**

To detect these mutations, we developed an effective technique based on the real-time TaqMan PCR system, which allows us to evaluate the copynumbers of the *PARK2 *gene exons by comparing the intensity of the amplification signals from some exon of this gene with that of the β-globin gene (the internal control).

**Results:**

We analyzed rearrangements in exons 1–12 of the *PARK2 *gene in 64 patients from Russia with early-onset Parkinson's disease. The frequency of these mutations in our patients was 14%.

**Conclusion:**

We have developed a simple, accurate, and reproducible method applicable to the rapid detection of exon rearrangements in the *PARK2 *gene. It is suitable for the analysis of large patient groups, and it may become the basis for a diagnostic test.

## Background

Parkinson's disease (PD) is one of the most common neurodegenerative disorders. It has a significant genetic component, particularly in individuals with early onset (younger than 50 years). To date, associations between three different genes/loci and early-onset Parkinson's disease (EOPD) have been identified: *PARK2*, which encodes parkin [1], *PARK6*, which encodes PINK1 [2,3], and *PARK7*, which encodes DJ-1 [4].

Parkin is expressed primarily in the nervous system and is one of the family of E3 ubiquitin ligases, which attach short ubiquitin peptide chains to proteins to tag them for degradation through the proteasomal pathway [5]. Different types of mutations, from point mutations to complex rearrangements, including deletions and multiplications of complete exons, have been described in the *PARK2 *gene [6-10]. Homozygous or compound heterozygous mutations in *PARK2 *are considered to cause the disease in approximately 50% of patients with the rare familial form, autosomal recessive juvenile PD [5,6,11]. The frequency of *PARK2 *mutations in patients with sporadic EOPD may depend on the inclusion criteria and ethnic background [12,13]. In general, parkin gene mutations are considered to be autosomal recessive. However, heterozygous mutations have been found to be associated with subclinical damage to dopamine metabolism in the striatum [14]. It is possible that heterozygous mutations in *PARK2 *can lead in some cases to early-onset or late-onset sporadic PD or to an increase in the risk of the disease, given interactions with other factors [15-17]. Therefore, it is necessary to analyze *PARK2 *mutations in a large group of patients with sporadic PD to test this hypothesis. One distinctive feature of the mutations in this gene is the high frequency (33%–67%) of exon rearrangements [7-9,18-20]. This type of mutation is only detectable by gene copy dosage assays. Here, we describe an effective and quantitative real-time polymerase chain reaction (PCR) method for the analysis of exon dosage in this gene.

## Methods

### Patients and samples

We studied two groups of patients: nine with autosomal recessive juvenile PD and 64 with apparent EOPD, with an age of onset of ≤ 50 years. All patients were examined clinically at the Institute of Neurology in the Russian Academy of Medical Sciences, Moscow. The diagnosis was confirmed using the UK Parkinson's Disease Brain Bank Criteria. All patients were assessed using a standard Unified Parkinson's Disease Rating Scale and Hoehn and Yahr scores [23]. All participants in this study gave their informed consent, and the study was approved by the local ethics committee. Venous whole blood was taken, and DNA was extracted from peripheral leukocytes by standard methods [24]. Deletions in *PARK2 *in the patients with juvenile PD were detected as described previously [10,17], and patients' DNA samples were used to optimize the efficiency of the real-time PCR method based on TaqMan^® ^technology (Applied Biosystems, Foster City, CA, USA).

### Exon dosage analysis

Primers and probes for exons 1–12 of *PARK2 *were designed using Vector NTI Suite 9 software (Invitrogen, Carlsbad, CA, USA) and the *PARK2 *sequence (GenBank: AB009973) (Table 1). The β-globin gene was coamplified with each individual exon of the *PARK2 *gene as the internal standard. Primers and probe sequences for the β-globin gene were as described previously [25] (Table 1). PCR was performed in 25 μL reaction volumes containing 10–20 ng of genomic DNA, 1 × PCR buffer (Syntol Corporation, Moscow, Russia), 2.5 mM MgCl_2_, 10 pM of the appropriate primers for the individual exons of *PARK2 *and the β-globin gene, 200 μM of each dNTP, 1.25 units of Hot-Rescue *Taq *DNA polymerase (Syntol Corporation, Moscow, Russia) and 4 pM of probes for the individual exons of *PARK2 *and the β-globin gene, designed using TaqMan chemistry (Syntol Corporation, Moscow, Russia). Amplification was performed for 2 min at 50°C, 10 min at 95°C, and 40 cycles of 15 s at 95°C and 50 s at the appropriate annealing temperature (Tann; see Table 1). The fluorescence intensity of the PCR products was measured using an ANA-32 machine (Institute for Analytical Instrumentation, St. Petersburg, Russia). All samples were tested in triplicate. We used the value of the cycle threshold (CT) to perform our calculations. The ANA-32 machine software determined the CT for every well by second-degree calculation. This method of CT determination is user independent and is based on a second-derivative value of the real-time fluorescence intensity curve. Because three different measurements were obtained per sample, the average CT (ΔCt) and standard deviation (SD) were calculated for both *PARK2 *and the β-globin gene. The internal control allowed the calculation of the relative ratio of *PARK2 *to the β-globin gene concentrations (R_p/b_). This was calculated using ΔCt according to the formula of Livak et al. [26]:

**Table 1:** Primers and probe sequences and annealing temperatures used.

**Gene/exon**	**Sequences**	**T_ann_**
β-globin	Forward primer: 5'-GTGCACCTGACTCCTGAGGAGA-3' Reverse primer: 5'-CCTTGATACCAACCTGCCCAG-3' Probe: 5'-*FAM*-AAGGTGAACGTGGATGAAGTTGGTGG-*RTQ1-3'*	60°C
PARK2\ex1	Forward primer: 5'-CAGCGGCTCTCCTGGGTTAAA-3' Reverse primer: 5'-TACGACTCCCAGCAGGCCCT-3' Probe: 5'-*ROX*-CCTAGGAATGCGCACGCGCGGAG-*RTQ1-*3'	60°C
PARK2\ex2	Forward primer: 5'-ATCTCAGGCATGAATGTCAGATT-3' Reverse primer: 5'-TGACCAGTTGCGTGTGATTT-3' Probe: 5'-*ROX*-ATGAGCAATGGAGCTGGCGGCATCC-*RTQ1*-3'	60°C
PARK2\ex3	Forward primer: 5'-CATTGTGCAGAGACCGTGGAGAA-3' Reverse primer: 5'-GCTGAGGTCCACCCGAGTCAA-3' Probe: 5'-*ROX*-AAATGAATGCAACTGGAGGCGACGACCC-*RTQ1*-3'	60°C
PARK2\ex4	Forward primer: 5'-TAGCCACTTCTTCTGCTTTT-3' Reverse primer: 5'-TGCACTGTACCCTGAGTTTT-3' Probe: 5'-*ROX*-ATTGCAAAGGCCCCTGTCAAAGAGTGCA-*RTQ1*-3'	60°C
PARK2\ex5	Forward primer: 5'-TCTTGCTGGGATGATGTTTTAATTCCAAA-3' Reverse primer: 5'-TTGCAATAAGAGGAATGAAT-3' Probe: 5'-*ROX*-AATGCCAATCCCCACACTGCCCTGG-*RTQ1*-3'	60°C
PARK2\ex6	Forward primer: 5'-CCGTGGAGGGAAGTGACACTAT-3' Reverse primer: 5'-TGCACCTGATCGCAACAAAT-3' Probe: 5'-*ROX*-TGTGCGCGTAATGCAAGTGATGTTCCG-*RTQ1*-3'	60°C
PARK2\ex7	Forward primer: 5'-GGGTCGTGAACAAACTGCCGATCATT-3' Reverse primer: 5'-AGGAGCCCCGTCCTGGTTTT-3' Probe: 5'-*ROX-*AAGCAAATCACGTGGCGGGAGTTGCAC-*RTQ1-*3'	60°C
PARK2\ex8	Forward primer: 5'-TCACTCACCTGCTCTTCTCCCAGAAT-3' Reverse primer: 5'-CAGAGTGAAAGTGACGTTTT-3' Probe: 5'-*ROX-*TCAAGGAGTTGGGACAGCCAGCTGTTGG-*RTQ1*-3'	60°C
PARK2\ex9	Forward primer: 5'-ACCCCACAGCCCAGGCCATT-3' Reverse primer: 5'-TGCAGATGGGGGGCGTGTTA-3' Probe: 5'-*ROX-*TTCGCAGGTGACTTTCCTCTGGTCAGGC-*RTQ1-*3'	60°C
PARK2\ex10	Forward primer: 5'-GCTTGGAGGAATGAGTAGGGCATT-3' Reverse primer: 5'-GTTTGCCTTCTGCCGGGAAT-3' Probe: 5'-*ROX-*AGGCTTCAAATACGGCACTGCACTCCCC-*RTQ1-*3'	60°C
PARK2\ex11	Forward primer: 5'-TGGTGGTTTTCTTGATGGTTT-3' Reverse primer: 5'-AACGCCTTTCCTCTTTGTTT-3' Probe: 5'-*ROX-*TCGGCGGCTCTTTCATCGACTCTGTAGG-*RTQ1*-3'	60°C
PARK2\ex12	Forward primer: 5'-CACGATCTTCCTGAGAAGTCAGACAAT-3' Reverse primer: 5'-ACAACACTGCATGCGGCAAA-3' Probe: 5'-*ROX*-TCTGCGTAGTGTGGGTAAGAGCATGCGG-*RTQ1*-3'	60°C

R_p/b _= 2 ^-(ΔΔCt)^

where ΔΔCt = [ΔCt *β-globin *(control sample) - ΔCt *PARK2 *(control sample)] - [ΔCt *β-globin *(patient sample) - ΔCt *PARK2 *(patient sample)]. All positive results were confirmed at least twice, and the average ratio was calculated. All samples with homozygous or heterozygous exon rearrangements affecting only one exon were confirmed with an independent set of primers to avoid any false positive results caused by primer mismatch associated with undetected polymorphisms.

## Results

We performed relative quantitative real-time PCR amplification based on TaqMan technology to analyze *PARK2 *exon dosage. With this method, it is possible to analyze the deletions and duplications of exons 1–12.

Exon dosage analysis was performed for nine genomic DNA samples from patients with autosomal recessive juvenile PD. These patients have been described elsewhere, and heterozygous *PARK2 *exon deletions have been identified in some of them [17].

The relative ratio of *PARK2 *to the β-globin gene concentration was calculated for all DNA samples (see Table 2). Based on the observed variability in the values of the ratios in normal individuals and in positive controls with heterozygous *PARK2 *rearrangements, we considered ratios between 0.7 and 1.3 as normal. Values lower than 0.6 or higher than 1.4 were interpreted as heterozygous deletions or duplications of the assessed exon, respectively. In the case of homozygous deletions, the ratio was 0.3 or lower.

**Table 2:** Results of exon dosage analysis for the seventeen previously described PD patients and controls.

Genotype	R_p/b _(mean ± SD)											
	
	Exon 1	Exon 2	Exon 3	Exon 4	Exon 5	Exon 6	Exon 7	Exon 8	Exon 9	Exon 10	Exon 11	Exon 12
Control	0,95 ± 0,08	0,97 ± 0,06	0,83 ± 0,10	0,99 ± 0,11	1,13 ± 0,05	1,11 ± 0,12	1,03 ± 0,07	0,95 ± 0,08	1,12 ± 0,09	0,98 ± 0,04	1,13 ± 0,05	1,09 ± 0,01
Control	0,88 ± 0,11	1,19 ± 0,10	0,86 ± 0,08	0,88 ± 0,03	0,90 ± 0,16	0,89 ± 0,10	1,00 ± 0,11	0,97 ± 0,04	0,89 ± 0,13	0,96 ± 0,01	0,99 ± 0,07	1,18 ± 0,03
Control	1,02 ± 0,03	1,12 ± 0,11	1,09 ± 0,19	1,03 ± 0,04	0,98 ± 0,06	0,90 ± 0,16	0,96 ± 0,10	1,01 ± 0,11	1,13 ± 0,02	0,98 ± 0,11	1,06 ± 0,07	1,02 ± 0,05
Patient without exon deletion	0,95 ± 0,04	0,83 ± 0,07	0,92 ± 0,10	0,97 ± 0,11	1,03 ± 0,10	1,2 ± 0,14	1,16 ± 0,06	1,07 ± 0,17	0,88 ± 0,12	1,04 ± 0,13	1,03 ± 0,05	1,11 ± 0,12
Patient without exon deletion	1,11 ± 0,02	0,82 ± 0,14	1,15 ± 0,03	1,16 ± 0,08	0,95 ± 0,01	0,96 ± 0,01	0,87 ± 0,05	1,04 ± 0,15	1,18 ± 0,09	0,98 ± 0,10	1,15 ± 0,10	1,03 ± 0,06
Patient without exon deletion	1,01 ± 0,05	0,90 ± 0,11	0,98 ± 0,13	0,89 ± 0,04	0,93 ± 0,06	1,19 ± 0,03	1,14 ± 0,08	1,03 ± 0,12	1,11 ± 0,14	1,10 ± 0,07	0,84 ± 0,11	0,89 ± 0,07
Patient with exon 2 heterozygous deletion	0,91 ± 0,03	**0,54 ± 0,05**	0,97 ± 0,10	0,92 ± 0,08	0,91 ± 0,06	1,01 ± 0,02	1,13 ± 0,01	1,16 ± 0,05	0,89 ± 0,08	0,82 ± 0,12	0,84 ± 0,13	0,90 ± 0,12
Patient with exon 3 heterozygous deletion	0,98 ± 0,05	1,12 ± 0,09	**0,66 ± 0,05**	1,13 ± 0,08	0,97 ± 0,07	1,00 ± 0,02	1,01 ± 0,12	0,97 ± 0,04	0,98 ± 0,05	1,12 ± 0,11	0,85 ± 0,13	0,90 ± 0,07
Patient with exon 4 heterozygous deletion	0,97 ± 0,02	0,95 ± 0,06	1,13 ± 0,08	**0,60 ± 0,08**	1,11 ± 0,15	0,86 ± 0,03	0,98 ± 0,09	0,91 ± 0,07	0,96 ± 0,05	1,08 ± 0,02	1,07 ± 0,03	1,08 ± 0,02
Patient with exon 5 heterozygous deletion	1,05 ± 0,02	0,95 ± 0,05	0,99 ± 0,15	1,19 ± 0,07	**0,60 ± 0,15**	1,02 ± 0,11	1,03 ± 0,09	0,85 ± 0,04	0,84 ± 0,08	0,98 ± 0,09	0,93 ± 0,03	1,03 ± 0,15
Patient with exon 6 heterozygous deletion	1,08 ± 0,01	1,04 ± 0,01	1,14 ± 0,07	0,97 ± 0,03	0,89 ± 0,09	**0,59 ± 0,04**	0,99 ± 0,03	1,01 ± 0,02	1,11 ± 0,01	1,00 ± 0,04	1,13 ± 0,02	0,96 ± 0,06
Patient with exon 7 heterozygous deletion	0,99 ± 0,09	0,97 ± 0,03	0,85 ± 0,02	0,88 ± 0,01	1,04 ± 0,08	1,2 ± 0,06	**0,58 ± 0,09**	1,09 ± 0,11	1,02 ± 0,02	1,05 ± 0,07	0,96 ± 0,03	0,93 ± 0,04

We examined 64 Russian patients with EOPD for exon rearrangements. The majority showed relative *PARK2 *exon to the β-globin gene ratios of approximately 0.8–1.2 for all exons under study. Alterations in gene dosage were found in nine patients (14%; Table 3). We detected a *PARK2 *exon 5 duplication in two patients (patients 8 and 9). Homozygous deletions were found in two: patients 2 (exon 4) and 5 (exon 3). Heterozygous deletion of a single exon was found in one patient (exon 3 in patient 6) and of several exons in four patients (patients 1, 3, 4, and 7; see Table 3). We could not distinguish heterozygous contiguous multiexonic deletions from compound heterozygous deletions. For precise genotype determination, it will be necessary to analyze additional intronic DNA markers or to study the parkin gene deletions in the relatives of patients.

**Table 3:** Results of exon dosage analysis for the nine PD patients with exon deletions and duplications.

Genotype	R_p/b _(mean ± SD)											
	
	Exon 1	Exon 2	Exon 3	Exon 4	Exon 5	Exon 6	Exon 7	Exon 8	Exon 9	Exon 10	Exon 11	Exon 12
Patient 1	0,98 ± 0,01	1,01 ± 0,17	**0,57 ± 0,02**	**0,55 ± 0,05**	**0,60 ± 0,15**	**0,59 ± 0,04**	**0,58 ± 0,09**	1,03 ± 0,07	0,98 ± 0,12	1,04 ± 0,02	1,06 ± 0,02	1,12 ± 0,10
Patient 2	1,03 ± 0,04	0,93 ± 0,14	1,05 ± 0,03	**0,01 ± 0,01**	0,95 ± 0,01	0,96 ± 0,02	0,88 ± 0,06	1,07 ± 0,02	0,89 ± 0,04	0,98 ± 0,03	1,06 ± 0,01	1,04 ± 0,02
Patient 3	1,08 ± 0,03	0,90 ± 0,04	**0,47 ± 0,03**	**0,61 ± 0,10**	0,93 ± 0,06	1,09 ± 0,03	1,11 ± 0,02	1,05 ± 0,04	1,11 ± 0,03	1,02 ± 0,06	0,94 ± 0,03	0,98 ± 0,03
Patient 4	1,00 ± 0,02	**0,54 ± 0,05**	**0,63 ± 0,02**	**0,60 ± 0,08**	0,91 ± 0,06	1,05 ± 0,11	1,03 ± 0,02	1,21 ± 0,05	0,94 ± 0,03	0,82 ± 0,14	0,88 ± 0,02	0,91 ± 0,12
Patient 5	0,99 ± 0,06	1,01 ± 0,08	**0,01 ± 0,02**	1,13 ± 0,08	0,97 ± 0,07	1,00 ± 0,02	1,06 ± 0,12	0,97 ± 0,04	1,02 ± 0,05	1,15 ± 0,04	0,87 ± 0,04	0,94 ± 0,03
Patient 6	0,89 ± 0,07	0,93 ± 0,02	**0,66 ± 0,04**	0,92 ± 0,03	1,11 ± 0,15	0,89 ± 0,04	0,93 ± 0,10	0,91 ± 0,08	1,13 ± 0,02	1,08 ± 0,01	1,07 ± 0,06	1,08 ± 0,01
Patient 7	0,86 ± 0,04	0,95 ± 0,12	**0,61 ± 0,09**	**0,63 ± 0,08**	**0,57 ± 0,12**	1,03 ± 0,10	1,06 ± 0,08	0,83 ± 0,05	0,94 ± 0,07	0,97 ± 0,02	0,98 ± 0,07	1,01 ± 0,11
Patient 8	1,02 ± 0,04	1,14 ± 0,01	1,01 ± 0,07	0,97 ± 0,03	**1,41 ± 0,16**	0,99 ± 0,04	0,99 ± 0,03	1,01 ± 0,06	1,01 ± 0,07	1,03 ± 0,08	1,02 ± 0,03	0,99 ± 0,02
Patient 9	1,21 ± 0,03	0,97 ± 0,10	0,93 ± 0,02	1,02 ± 0,06	**1,43 ± 0,17**	1,02 ± 0,06	1,13 ± 0,01	1,01 ± 0,12	1,11 ± 0,03	1,03 ± 0,08	0,98 ± 0,04	0,96 ± 0,05

## Discussion

Here, we describe a cost-effective technique for the rapid and accurate detection of exon rearrangements in the *PARK2 *gene, using a real-time TaqMan PCR system. Joint amplification of individual *PARK2 *exons and the β-globin gene as the internal control and the use of the least-squares method to calculate the cycle threshold ensured the adequacy of the analysis and the repeatability of the method. For method verification, we examined nine patients with juvenile autosomal recessive PD whose deletion status determined earlier (three patients without deletions and six with monoexonic heterozygous deletions). We also studied 64 Russian patients with EOPD and found exon rearrangements in nine of them using this method. Our method is suitable for the rapid detection and screening of exon rearrangements in the parkin gene. However, for heterozygous contiguous deletions of neighboring exons, it cannot distinguish between a large contiguous deletion on a single chromosome and a combination of two deletions on both chromosomes. This method is not applicable to the detection of point mutations or small sequence changes.

To date, several studies have addressed the question of exon dosage in *PARK2 *using different approaches [7,8,20-22]. Lucking et al. [7] performed the first screening to determine the exon dosage based on semiquantitative multiplex PCR. However, the amounts of PCR products were measured in the putative log-linear phase of their multiplex PCR without consideration of the constant amplification efficiency of all primer pairs. Other previously described methods for the detection of the *PARK2 *gene exon dosage and the method described here are based on real-time PCR, which provides a precise measurement of the cycle threshold [8,20-22]. One such assay was based on an intercalating dye (SYBR Green I), which functions as a fluorescent reporter, and used unlabeled primers [20]. Because that method uses only a single fluorescent reporter, two independent amplifications (of *PARK2 *and the β-globin gene) are necessary for each DNA sample. However, application of the LightCycler (Roche Applied Science, Indianapolis, IN, USA) or TaqMan system allows us to carry out only one multiplex amplification reaction for each DNA sample [8,21,22]. This improves sensitivity and repeatability and reduces the time taken. Here, we used a combination of ROX-labeled parkin gene and FAM-labeled β-globin gene probes in a single-tube multiplex PCR amplification. To avoid problems of the possible loss of signal when there are variations within the binding sites, we selected probes with low melting temperatures. The annealing temperature we used was 60°C. In the case of a mismatch, such probes will still generate a signal.

Both the TaqMan and the LightCycler systems allow the application of quantitative real-time PCR assays by using labeled oligonucleotide probes, which generate sequence-specific signals. In the TaqMan system, only one probe is used for each locus, whereas in the LightCycler system, two hybridization probes are necessary, so it is more complicated.

Two other groups have measured exon dosage based on TaqMan technology [21,22]. Maruyama et al. [21] performed a PCR assay with TaqMan probes but only for exons 1–5. However, rearrangements in other exons (7–12) have been described in some populations of patients with PD [10,13,15,17]. Thus, the determination of those deletions and duplications is very important for diagnostic purposes. Sinha et al. [22] used an 84-bp fragment of a single-copy human β-actin gene as the internal control. However, in that technique variant, a standard curve of human genomic DNA concentrations must be generated for every PCR run [22], so it is more complicated and less repeatable than our method.

All these methods are based on different types of real-time PCR. Recently, a new approach has been described, called multiplex ligation-dependent probe amplification (MLPA). MLPA is based on the combination of a ligation reaction and standard PCR followed by electrophoresis. It has been shown that this method is also applicable to exon dosage analysis [27]. However, a direct relationship between exon dosage and the amount of the ligation reaction product has not yet been proved. Thus, MLPA can be considered only as a semiquantitative method and not as an alternative to real-time-PCR-based methods. In our opinion, it would be ideal to combine real-time TaqMan PCR and MLPA to confirm the results further.

## Conclusion

This TaqMan real-time PCR methodology can be applied to the rapid and precise screening of exon rearrangements in the *PARK2 *gene in patients with PD. It analyzes all relevant deletions and duplications of exons 1–12. The determination of the TaqMan-based cycle threshold is more precise than earlier methods based on SYBR Green I labeling. A very precise measurement of exon dosage is possible using a second-derivative value of the real-time fluorescence intensity curve. This method is applicable to the analysis of large patient groups and may become the basis for a diagnostic test.

## List of abbreviations

PD: Parkinson's disease

EOPD: early-onset Parkinson's disease

PCR: polymerase chain reaction

CT: cycle threshold

SD: standard deviation

## Competing interests

The author(s) declare that they have no competing interests.

## Authors' contributions

MIS and EVS designed and executed all the experiments. MIS and EVS analyzed the data and wrote the paper. GHB, SNI and III-S participated in the acquisition of data and helped draft the manuscript. PAS and SAL were involved in study design and helped draft the manuscript. All authors read and approved the final manuscript.

## Pre-publication history

The pre-publication history for this paper can be accessed here:


